# DSA-net: a lightweight and efficient deep learning-based model for pea leaf disease identification

**DOI:** 10.3389/fpls.2025.1642453

**Published:** 2025-08-28

**Authors:** Laixiang Xu, Yiru Duan, Zhaopeng Cai, Wenwen Huang, Fengyan Zhai, Junmin Zhao

**Affiliations:** ^1^ School of Computer and Data Science, Henan University of Urban Construction, Pingdingshan, China; ^2^ School of Computer and Data Science, Research Center of Smart City and Big Data Engineering of Henan Province, Henan University of Urban Construction, Pingdingshan, China; ^3^ School of Agricultural Resources and Environment, Henan Institute of Science and Technology, Xinxiang, China; ^4^ Department of Plant Pathology, Henan Province Engineering Research Center of Biological Pesticide and Fertilizer Development and Synergistic Application, Henan Institute of Science and Technology, Xinxiang, China

**Keywords:** leaf disease, pea leaf, deep learning, MobileNet-V3_small, attention mechanism

## Abstract

**Introduction:**

Pea is a nutrient-dense, functionally diversified vegetable. However, its leaf diseases have a direct impact on yield and quality. Most approaches for identifying pea leaf diseases exhibit low feature extraction efficiency, significant environmental sensitivity, and limited large-scale applications, making it impossible to meet the expectations of modern agriculture for accuracy, real-time processing, and low cost.

**Methods:**

Therefore, we propose a deep learning model for pea leaf disease identification based on an improved MobileNet-V3_small, deformable convolution strategy, self-attention, and additive attention mechanisms (DSA-Net). First, a deformable convolution is added to MobileNet-V3-small to increase the modeling skills for geometric changes in disease features. Second, a self-attention mechanism is integrated to improve the ability to recognize global features of complex diseases. Finally, an additive attention strategy to enhance the feature channel and spatial position response relationship in edge-blurred lesion areas. The experimental pea leaf data set consists of 7915 samples divided into five categories. It includes one healthy leaf and four diseases: brown spot, leaf miner, powdery mildew, and root rot.

**Results:**

The experimental results indicate that the suggested DSA-Net has an average recognition accuracy of 99.12%. It has a parameter size of 1.48M.

**Discussion:**

The proposed approach will help with future edge device deployments. The current proposed technique considerably enhances the diagnostic accuracy of pea leaf diseases and has significant promotion and application potential in agriculture.

## 1 Introduction

Due to their high protein, vitamin, and mineral content, peas are one of the most important leguminous crops in the world and a necessary part of the human diet (Wu et al., 2023). Peas are important to agriculture because of their wide range of applications and high level of environmental adaptability. However, throughout their growth stage, peas are especially susceptible to a number of leaf diseases, such as powdery mildew, brown spot, and leaf miner. Plant quality, productivity, and healthy growth can all be directly impacted by pea leaf diseases. In severe cases, they may even lead to crop failure, which would cause agricultural output to suffer large financial losses.

Traditional techniques of plant leaf disease identification rely mostly on personal observation and empirical judgment, which are sometimes supplemented with simple diagnostic equipment. This strategy is not only inefficient, but it is also heavily dependent on expert expertise, which is very subjective. It is typically appropriate for small farmers or preliminary screening, but it is difficult to meet the actual requirements of large-scale agricultural production. Due to subjectivity, lag, low precision, and low efficiency, it is prone to misdiagnosis, delayed prevention and treatment, or overuse of medication. With the development of computer technology, some machine learning methods based on simple image features (Deng et al., 2020) have been applied to disease recognition. For example, Ali et al. (2022) proposed a method called feature fusion and principle component analysis, which has an accuracy of 98.2%. Paymode and Malode (2022) employed a convolutional neural network (CNN)-based visual geometry group (VGG) model to enhance performance measurement. In the trial, the proposed approach had an average accuracy of 97.06% for grapes and tomatoes leaf diseases. However, classic machine learning methods based on simple picture features have clear drawbacks in feature expression, environmental adaptation, real-time performance, making it impossible to satisfy the objectives of modern agriculture for accurate and efficient disease recognition (Dang et al., 2024).

In recent years, deep learning technology has demonstrated strong performance advantages in the field of image recognition, and this technological innovation is driving the rapid development of agricultural disease recognition towards intelligence and precision. Deep learning models can automatically learn advanced features of images, which has significant advantages in improving recognition accuracy and efficiency. This advantage is gradually replacing traditional image processing methods and becoming the mainstream technical solution in the field of intelligent recognition of agricultural diseases (Kanda et al., 2022). Among them, the MobileNet series models, with their lightweight characteristics (Srinivasu et al., 2021), have a good balance between recognition accuracy and computational efficiency, making them more suitable for the application needs of real-time diagnosis scenarios in the field. The core modules of MobileNet-V3_small include the inverted residual block, the squeeze and excitation module, and the h-swift activation function. The h-swift activation function, the squeeze and excitation module, and the inverted residual block are the three key components of MobileNet-V3_small. Utilizing cutting-edge concepts like depthwise separable convolution and inverse residual structure, MobileNet-V3_small significantly lowers computation costs and parameter counts without sacrificing accuracy. When it comes to identifying pea leaf diseases. Due to some current models’ limitations in dynamic feature extraction (e.g., small targets/occluded scenes), the static weight allocation of depthwise separable convolutions is difficult to adapt to spatially changing features.

In response to the above shortcomings, this study aims to improve the accuracy and robustness of the MobileNet-V3_stall model in identifying pea leaf diseases. Specifically, this study will introduce deformable convolution network (DCN) and self-attention (SA) and additive attentive (AA) mechanisms to enable the model to pay more attention to the key features of disease areas. Through these improvement strategies, a recognition accuracy of 99.12% was achieved on a self-built dataset, which is 10.5% higher than the baseline model. The main contributions of this paper are as follows:

1. The deformable convolution adjusts the convolution kernel’s sample location by adding offset. It accurately captures the varied morphological aspects of pea leaf diseases and responds effectively to geometric changes.
2. The self-attention mechanism automatically learns feature position correlations, improves important local feature extraction, and strengthens the model’s ability and robustness to deal with complex relationships between diseased and healthy regions.
3. The additive attention mechanism dynamically distributes channel and spatial weights. It highlights important disease features and effectively suppresses noise interference.
4. The suggested method retains the model’s lightweight architecture. It ensures efficient operation on resource-constrained edge devices and provides feasibility for real-time and accurate identification of pea leaf diseases.

We incorporated the DCN, SA, and AA modules individually in MobileNet-V3 for the first time, achieving collaborative optimization of capturing morphology+position+scale+small lesion enhancement rather than adding attention modules in isolation. Especially DCN has a unique ability to capture local features. It can adaptively adjust the shape and position of the convolution kernel based on the content of the input image, thereby focusing more accurately on the small feature changes in the lesion area, which is difficult to achieve with traditional fixed convolution kernels. The self-attention mechanism is adept at capturing long-range dependencies in images and can automatically identify associations between different regions. The additive attention mechanism further enhances the model’s ability to allocate weights to different features, and it can dynamically adjust the contribution of each feature according to task requirements. These three modules work together, with the DCN providing fine local features, the self-attention mechanism constructing global correlation information, and the additive attention mechanism optimizing feature weights, resulting in a powerful combination of complementary functions that differs significantly from existing methods that use only one attention mechanism or simply overlay convolution and attention mechanisms.

Our suggested model exhibits a significant degree of architectural foresight. In particular, we shall effectively and profoundly combine additive attention, self-attention, and DCN. distinct from some of the basic component-mixing techniques now in use. Here, we adopt a hierarchical interactive architecture. In the shallow layer of the basic model MobileNet-V3, we use DCN to perform preliminary feature extraction on the input image and obtain local detail information. We feed these features into the self-attention module to create an association of global feature information. At the same time, we employ an additive attention mechanism to dynamically alter the weights of features at each level, ensuring that the features are constantly tuned during the transmission process. This hierarchical dynamic architecture allows the model to fine-tune features at various levels, maximizing the benefits of each component and achieving more efficient feature extraction and classification. Compared to typical single architectures or simple combination architectures, our architecture is more adaptable to illness classification tasks, with improved performance and stronger generalization capacity.

The proposed model is capable of accurately comprehending the intricate patterns of disease features and making prompt and precise decisions based on the distinct process of disease classification. The integrated model we propose has a unique operating mechanism. It can adapt well to this demand. Specifically, DCN provides rich initial information for the model by accurately capturing local features, just like building a detailed ‘disease feature map’ for the model. The self-attention mechanism can search for key “paths” on this map, establish connections between different features, and form a global “disease feature network.” The additive attention mechanism assigns an “importance score” to each feature based on the information in this network, highlighting the most helpful features for classification. This mechanism enables the model to quickly and accurately identify key features of diseases and classify them based on these features.

Overall, our core work is to propose and validate a high-precision identification and optimization network framework for pea leaf diseases in device resource-constrained scenarios. We achieve a good combination of model efficiency and identification accuracy by combining lightweight design with two complementary attention methods based on MobileNet-V3, as well as customizing integration and collaboration at key layers. The parameters of the optimized model are as low as 1.48M. It is easy to deploy and run efficiently on edge computing platforms such as UAVs to realize real-time detection of field diseases. This efficient and practical solution for practical agricultural applications, such as drones and mobile terminals, provides better technical references for the deployment of this field and has direct application prospects and practical significance.

## 2 Literature review

Crop disease detection is an important activity in agricultural production and an essential component of image processing. However, it confronts numerous obstacles, such as complicated backdrops, high similarity, and difficulty in region segmentation (Kennedy and Huseth, 2022). The approaches utilized for crop disease and pest identification are currently classified into two categories: traditional machine learning and mainstream deep learning algorithms (Jafar et al., 2024).

We summarized the traditional machine learning (ML), deep learning (e.g., EfficientNet and VGG), and attention-based models and compared their strengths and weaknesses. The results are presented in **Table 1**.

**Table 1:** Comparison of different methods.

Technical category	Methods	Advantages	Limitations	Relevance to this Study
ML	SVM+PCA and AdaBoost	Small parameter count, suitable for edge deployment	Relying on manual features	Highlighting the necessity of automatic feature learning
Basic CNN	VGG and AlexNet	End to end training with high accuracy	High computational cost	Comparison baseline for lightweight design
Lightweight CNN	MobileNetV3 and EfficientNet	Balancing efficiency and accuracy	Poor adaptability to geometric deformation of lesions	Improved target for deformable convolution
Attention Model	Vision Transformer	Dependency modeling	Difficulty in edge deployment	Global attention hierarchical design

Traditional machine learning algorithms are challenging to apply to disease severity assessments (Huang et al., 2021). For example, Gutierrez and Goodwin, (2022) presented a cutting-edge molecular approach for detecting wheat pathogens based on loop-mediated isothermal amplification. Kumar and Kannan (2022) designed an adaptive boosting support vector machine (SVM) classifier to detect rice diseases such as bacterial leaf blight, brown spot disease, and leaf rust. This classifier detects and classifies rice leaf diseases with an accuracy of up to 98.8%. To detect leaf-based diseases, Reddy et al. (2021) employed the support vector machine (SVM) and the random forest (RF) approach. Bao et al. (2021) created a new approach for diagnosing wheat leaf diseases and their severity that uses the elliptical maximum interval criteria metric learning and has an accuracy rate of 94.16%. These methods have the advantage of being suitable for edge device deployment (e.g., SVM and RF). However, there are several important disadvantages, such as the reliance on manual feature extraction, low generalization ability for small samples, and poor adaptability to complicated backdrops.

Deep learning-based image processing and classification techniques have become popular research methodologies in both academia and industry as artificial intelligence research advances. Using the YOLOv4 algorithm and Plant Village’s 50000 multi-species leaf dataset, together with data augmentation, Aldakheel et al. (2024) proposed a 99.99% disease diagnostic accuracy. By merging modules and adjusting parameters, Sharma et al. (2023) used the DLMC Net lightweight convolutional network to achieve detection accuracy of 93.56%-99.50% in cross-crop disease detection with a parameter scale of 6.4 million. By building a dataset of 5932 rice leaves and integrating deep learning and transfer learning, Ritharson et al. (2023) suggested a customized VGG16 model that effectively recognized nine different disease categories with an accuracy of 99.94%. Haridasan et al. (2023) proposed an automated method that combines computer vision, image processing, machine learning, and deep learning techniques to construct a rice field disease recognition system. The system integrates support vector machine classifiers and convolutional neural networks for disease classification and achieves a maximum validation accuracy of 91.45% using ReLU and Softmax functions. Elaraby et al. (2022) proposed a deep learning method for detecting 25 different plant diseases, and the deep convolutional neural model AlexNet achieved an accuracy of 98.83%. Although deep learning methods have made significant progress in plant leaf disease recognition, research needs to shift from laboratory accuracy to field robustness and focus on solving the collaborative optimization problem of data, models, and applications.

## 3 Proposed methods

### 3.1 Introducing deformable convolution

In the task of identifying pea leaf diseases, MobileNet-V3_stall has the advantages of lightweight architecture and efficient computation. Therefore, we used the lightweight convolutional neural network MobileNet-V3_small as the basis model for the recognition of pea leaf diseases. However, the classic MobileNet-V3 failed to maintain a balance between shape adaptive feature extraction, global local feature collaborative optimization, and computational efficiency This results in unsatisfactory recognition performance. Therefore, we improved its network architecture.

MobileNetV3’s depth-separable convolution produces a predetermined geometric distribution pattern of receptive fields using kernel sampling based on a regular rectangular grid design. Images of diseased leaves can show intricate morphological changes. For example, the size, form, and position of the lesion may change, and traditional convolution’s fixed geometric structure may fail to capture these changes. Additionally, there is the issue of occlusion. Leaf photos taken in natural settings may be partially hidden by neighboring leaves or soil. When dealing with occlusion, traditional convolution may lose some key properties. As a result, MobileNet-V3_stall’s intermediate and shallow layers (layers 3, 4, and 8) now have deformable convolution. By learning the offset, the convolution kernel focuses on unobstructed areas, increasing feature extraction flexibility, discriminability, and balancing detail capture with computing efficiency.

In terms of feature abstraction hierarchy and adaptability to the DCN, the shallow layers 1 and 2 of the MobileNet-V3 mainly extract low-level features such as edges and textures, with low spatial deformation. Conventional convolution is sufficient. The middle layers 3 and 4 of the MobileNet-V3 begin to capture object components and local structures (such as lesion contours and region boundaries). The deformation characteristics of the DCN can adapt to irregular geometric shapes and improve feature expression ability. Compared to shallow layers (1–2 layers), introducing the DCN in layers 3 and 4 can bring more significant feature enhancement effects. Compared to deeper layers, it does not affect overall efficiency due to excessive computational complexity. The deep layer 8 of the MobileNet-V3 is approaching the output layer of the network. Although the features are more abstract at this point, the computational cost of introducing the DCN will further increase with the depth of the network. As the features of the 8th layer are crucial for the final classification task. Introducing the DCN in the layer 8 can to some extent uncover deep and potential feature interaction patterns in the data, improving the performance of the model. Moreover, by reasonably controlling the application scope of the DCN, it can find a balance between computational cost and performance improvement.

In terms of balancing computational complexity and model performance, the computational complexity of the DCN mainly comes from two aspects: firstly, the need to calculate offset for each sampling point, which adds additional parameters and computational complexity. During the convolution process, interpolation operations need to be performed based on the offset to obtain feature values on irregular grids, which also incurs additional computational overhead. In lightweight models like the MobileNet-V3, there is a trade-off between computational efficiency and model performance. If we apply the DCN to all layers of the MobileNet-V3, it will result in a significant increase in computational complexity, making it difficult for the model to run efficiently in resource- constrained environments such as mobile devices. And we chose to introduce the DCN in layers 3, 4, and 8, taking into account the importance of each layer’s features and computational costs before making the decision. These layers play a crucial role in feature transformation and expression in the model. In summary, introducing the DCN can effectively improve the performance of the model without significantly increasing the overall computational complexity of the MobileNet-V3.

**Figure 1:**
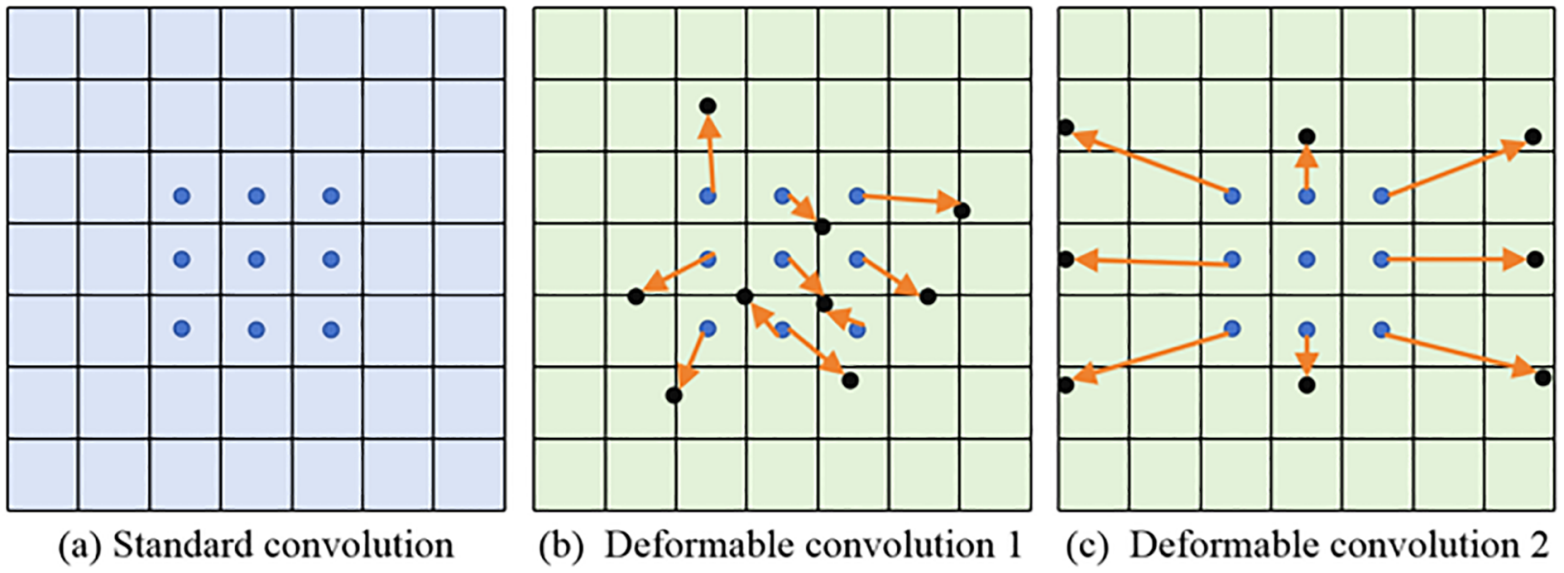
The comparison of standard and deformable convolution.

The DCN has strong geometric adaptability and can learn more useful information about the target object. The comparison of deformable and standard convolution is revealed in **Figure 1**.

**Figure 2:**
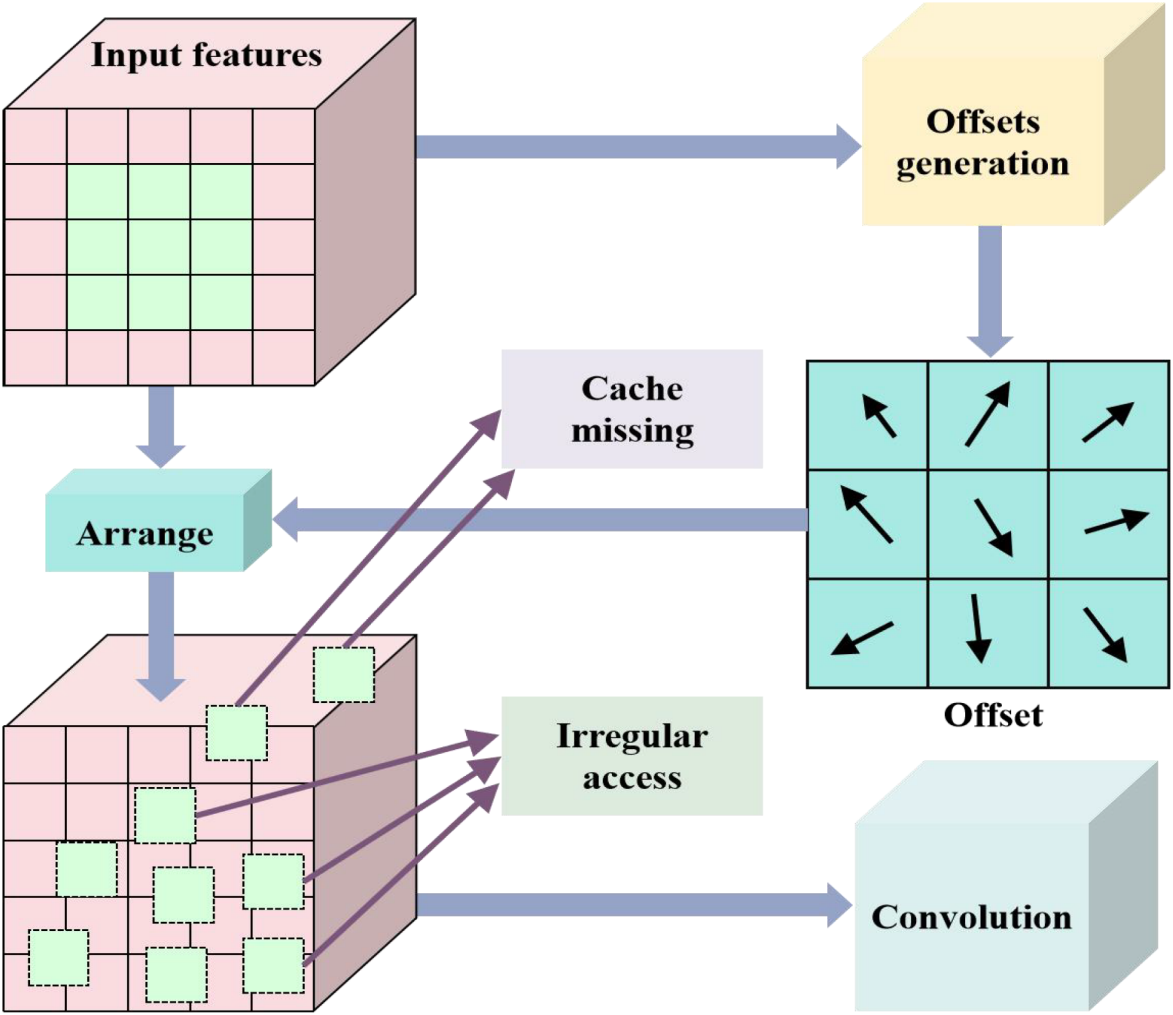
Proposed deformable convolution.

Some important observations can be made from the content of **Figure 1**. The deformable convolutions add an offset to each convolution sampling point in ordinary convolutions. By dynamically learning the offset of sampling points, the size and position can be used to identify object deformations based on geometric shapes. After increasing the offset, the flexible sampling mechanism of capturing the geometric features of irregular targets more accurately enables the convolution kernel to fit the object contour like an amoeba, significantly improving its modeling ability for complex visual patterns such as deformation and rotation. The proposed deformable convolution is sketched in **Figure 2**.

In **Figure 2**, the expression of the offset generation can be defined Equation (1):


(1) $$\Delta pk=f_{\text{offset}}(x)$$

where $f_{offset}$ is that the offset generation learns the offset through auxiliary convolution

After deformation, the sampling can be expressed Equation (2):


(2) $$y(p)=\sum_{K=1}^{K}w_{k}•x(p+p_{k}+\Delta p_{k})$$

where $p_{k}+\Delta p_{k}$ is the sampling position after dynamic adjustment.

Offset learning updates the parameters of the offset generation network through gradient back propagation. It can be calculated Equation (3):


(3) $$\frac{\partial L}{\partial \Delta p_{k}}=\sum_{p}\frac{\partial L}{\partial y(p)}•w_{k}•\frac{\partial (xp+p_{k}+\Delta p_{k})}{\partial \Delta p_{k}}$$

where $\frac{\partial L}{\partial \Delta p_{k}}$ is the gradient of the offset $\Delta p_{k}$ of the loss function *L* to the *k*-th sampling point. The function is to guide how to adjust the parameters of the offset generation network to make the sampling points more suitable for the deformation of pea leaves. where $\frac{\partial L}{\partial y(p)}$ is the gradient of the loss function at position *p* with the output characteristic graph *y*, reflecting the importance of this position for the classification of pea leaf lesions. $w_{k}$ represents the weight of the *k*-th sampling of the convolution kernel to weight the gradient signal. The weight of the convolution kernel with high importance will amplify the adjustment amplitude of the offset.

We used the variable convolution to replace the 3×3 convolution in the inverse residual block. Deep separable convolution of the classical MobileNet-V3 can be expressed Equation (4):


(4) $$y=Conv_{1*1}(DepthwiseConv_{3*3}(x))$$

The improved expression can be formulated Equation (5):


(5) $$y=Conv_{1*1}(DeformableDepthwiseConv_{3*3}(x))$$

### 3.2 Integrating self-attention

**Figure 3:**
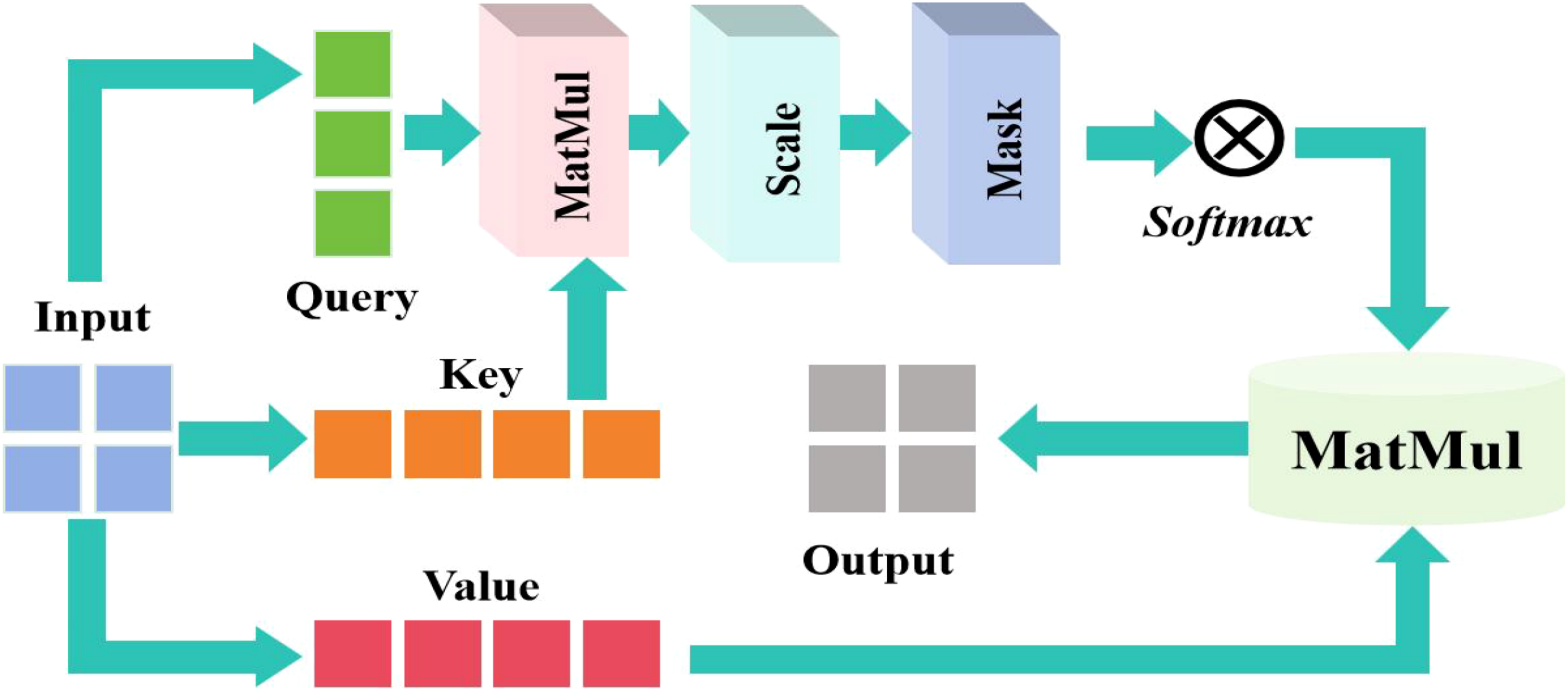
Integrated self-attention mechanism.

Although adding deformable convolution to the classical MobileNetV3 improved the model’s modeling ability for non-rigid geometric deformations as well as the robustness of complex deformation scenes in tasks like leaf recognition, the adaptive adjustment of its local receptive field may ignore global contextual information, resulting in insufficient capture of subtle deformations or distant feature associations. To compensate for the limitations of global information modeling, I added a self-attention mechanism to the sixth and tenth layers, which creates long-range dependency links. The self-attention system is involved in adaptively weighting color-related regions, increasing dominating colors via global linkages, and reducing noise during recognition. The integrated self-attention mechanism is manifested in **Figure 3**.

In the self attention mechanism, the input sequence is $X=[x_{1},x_{2},\cdot \cdot \cdot ,x_{n}]$ . The linear transformation generates Q, K, and V through the weight matrix $W^{Q}$ , $W^{K}$ , and $W^{V}$ . They can be described Equations (6)–(8):


(6) $$Q=XW^{Q}$$

(7) $$K=XW^{K}$$

(8) $$V=XW^{V}$$

where *Q* is the color characteristics of the current area. It focused on the suspected lesion area. *K* means to provide a feature library to be compared, that is, to store the global features of blades. *V* contained actual feature information and carries disease-specific features. Then use the attention weight to do softmax for the score, and finally use the weight to sum the *V* weight to map the features to the specific lesion label to complete the classification task.

The calculation formula of the proposed self-attention can be succinctly expressed Equation (9):


(9) $$A(Q,K,V)=F_{soft\max}(\frac{QK^{T}}{\sqrt{d_{k}}})$$

where $d_{k}$ s the dimension of the key. The softmax function is used to normalize the attention weight into a probability distribution, so as to express the importance of each time step.

### 3.3 Fusing additive attention

Although the effect of integrating the self-attention module is evident, self-attention can collect global information, but the computational complexity is substantial, particularly on high-resolution feature maps, which may result in a significant computational overhead. Furthermore, in some situations, self-attention may focus too much on the global and miss local details or fail to efficiently combine features at multiple levels, and noise may disrupt the global weights of self-attention. Therefore, we added additional attention. First, additive attention dynamically alters feature weights to help the model focus on relevant regions and improve detail capture capabilities. Second, additive attention strengthens the model by explicitly describing the relationship between significant features while suppressing irrelevant noise. Compared to self-attention, additive attention has the ability to dynamically suppress noise, optimize computational efficiency, and model stronger local features. It provides a mix of accuracy and speed in recognizing pea leaf diseases through dynamic feature calibration and efficient computing, making it ideal for lightweight deployment in complex situations. As a result, we applied additive attention to the 7th and 9th layers of this network. This modification allows the model to strike a better balance between lightweight, accuracy, and resilience, making it ideal for resource-constrained edge device deployment in agricultural applications. The model’s adaptation to complex agricultural situations is considerably improved by a joint design of self-attention (i.e., global modeling) and additive attention (i.e., local optimization), while maintaining lightweight properties. This hierarchical attention architecture provides a cost-effective technical path for precision agricultural diagnosis in resource-constrained environments, especially suitable for edge deployment scenarios that require balancing computational costs and recognition accuracy.

**Figure 4:**
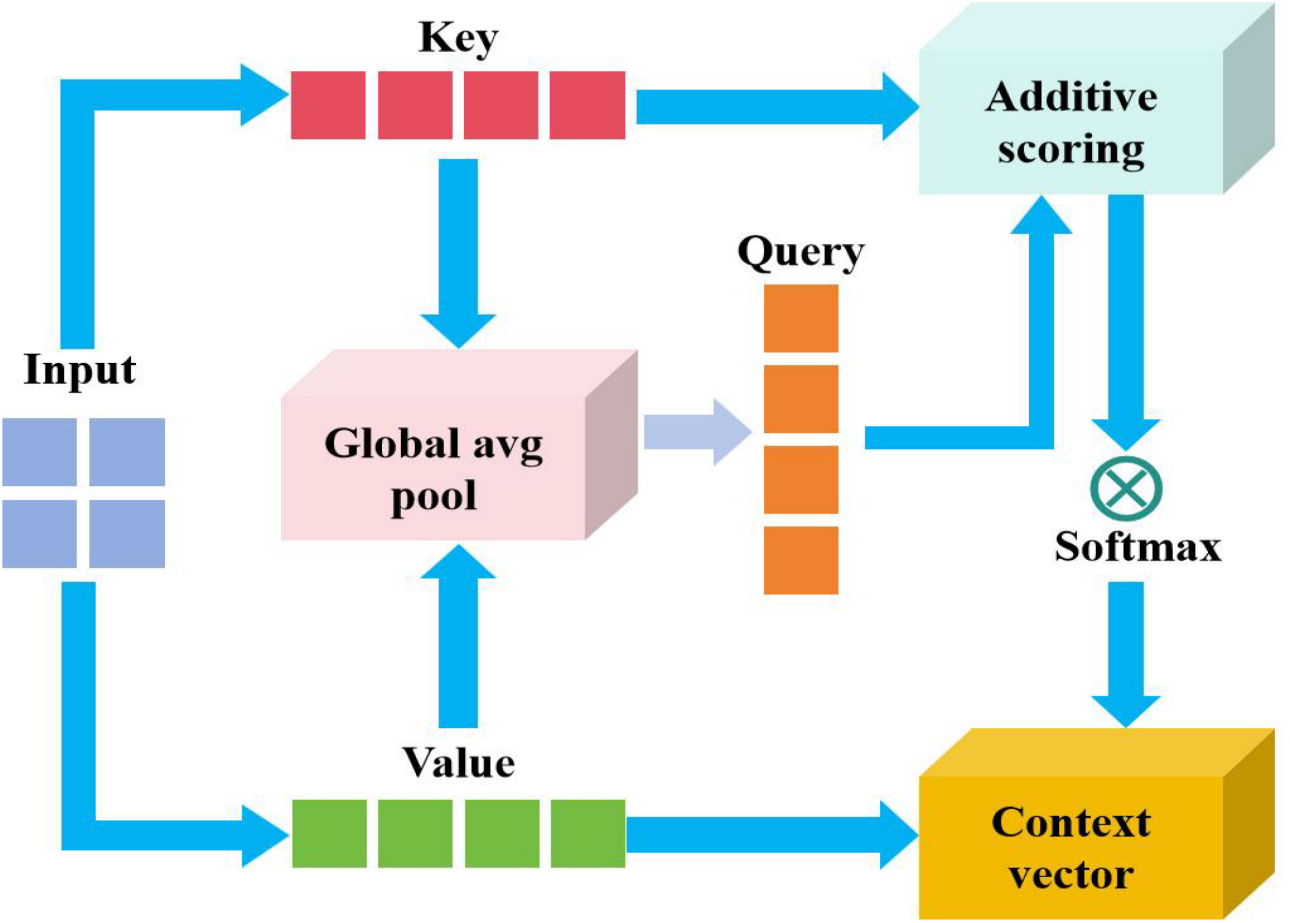
Proposed additive attention mechanism.


The proposed additive attention strategy is revealed in **Figure 4**. The key elements in the diagram can be decomposed into Keys(K) and Values(V). From the input feature representation, K is used to calculate the attention weight. V is the actual weighted feature, and finally generates the context quantity. The matching degree between query and key is calculated through addition operation. The formula can be expressed Equation (10):

(10) $$Score(Q,K)=V^{T}\tanh(W_{q}Q+W_{q}K)$$

where, $W_{Q}$ and $W_{K}$ are the learnable weight matrix and *v* is the projection vector.

The score is normalized by softmax to obtain the weight distribution. It can be defined Equation (11):


(11) $$\alpha_{i}=Soft\max(Score(Q,K))$$

Aggregation characteristics after weighted sum of weights and values. It can be calculated Equation (12):


(12) $$C=\sum_{i}\alpha_{i}V_{i}$$

The input characteristics are split into K (key) and V (value), and the two dimensions are usually the same or related. The matching degree between the query (such as the current decoder state) and each key is calculated through the additive scoring function. Normalize the score result with softmax to obtain the attention weight (i.e., highlight important features). The values are weighted and summed with weights, and the fused context vector C is output.

**Figure 5:**
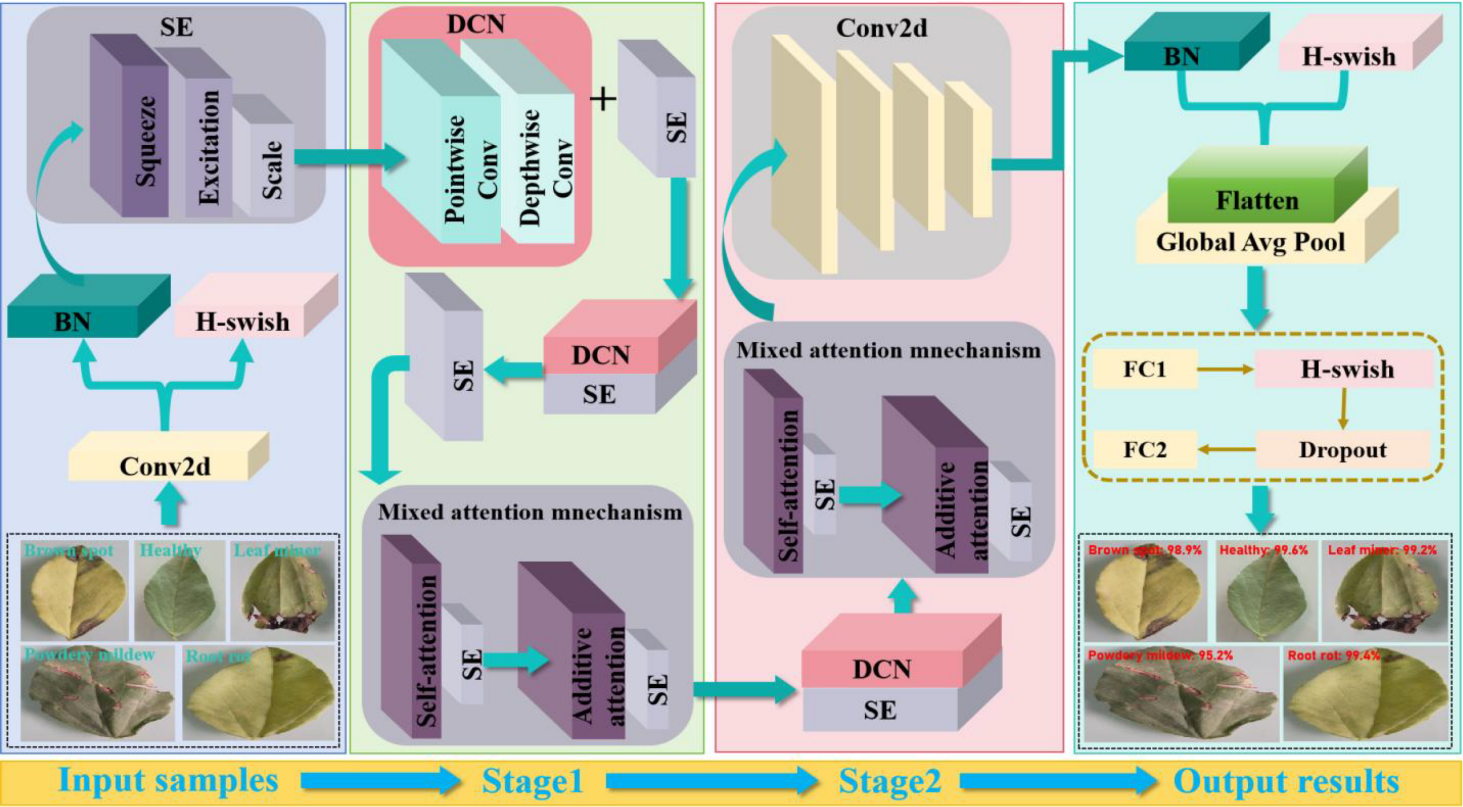
Proposed overall framework DSA-Net for pea leaf disease identification.

Based on the above analysis, the overall framework DSA-Net of pea leaf disease identification is presented in **Figure 5**. In the proposed model design, the positioning of SA and AA modules is determined based on improving model performance and optimizing feature extraction. Specifically, the SA enables the model to focus on the correlation between different parts of the data when processing input data, thereby better capturing global information. The SA can make the model focus on the associations between different regions in the image, which helps to understand the overall semantics of the image. The reason why we chose to apply the SA before the AA is that the SA can first perform global feature correlation analysis on the input data. This provides a more comprehensive and global perspective feature representation foundation for subsequent attention processing. Through the SA processing, the model has gained a certain understanding of the internal structure and relationships of the data. At this point, the AA module can be used to fine-tune attention to certain tasks or features. In the task of identifying pea leaf diseases, the SA can first direct the model to focus on the correlation between distinct disease areas of peas and determine the overall morphological properties of the leaves. Next, the AA module can focus more accurately on the disease characteristics, highlighting the lesion areas on the leaves. Therefore, applying hierarchical processing such as the SA before the AA can fully leverage the advantages of different attention mechanisms and improve the model’s ability to recognize complex features.

## 4 Experimental results and analysis

### 4.1 Experimental setup

We established a corresponding experimental platform. **Table 2** provides a detailed list of the operating system, graphics card, central processing unit, memory, deep learning framework, editor, and programming language used in the experiment.

**Table 2:** The experimental setup list.

Operating system	Windows 11(64-bit)
Graphics Card	NVIDIA GeForce RTX 4090
Central Processing Unit	i9-13900HX
Memory	32GB
Deep learning framework	Pytorch 2.4.1
Editor	PyCharm 2024.1
Programming language	Python 3.12

**Figure 6:**
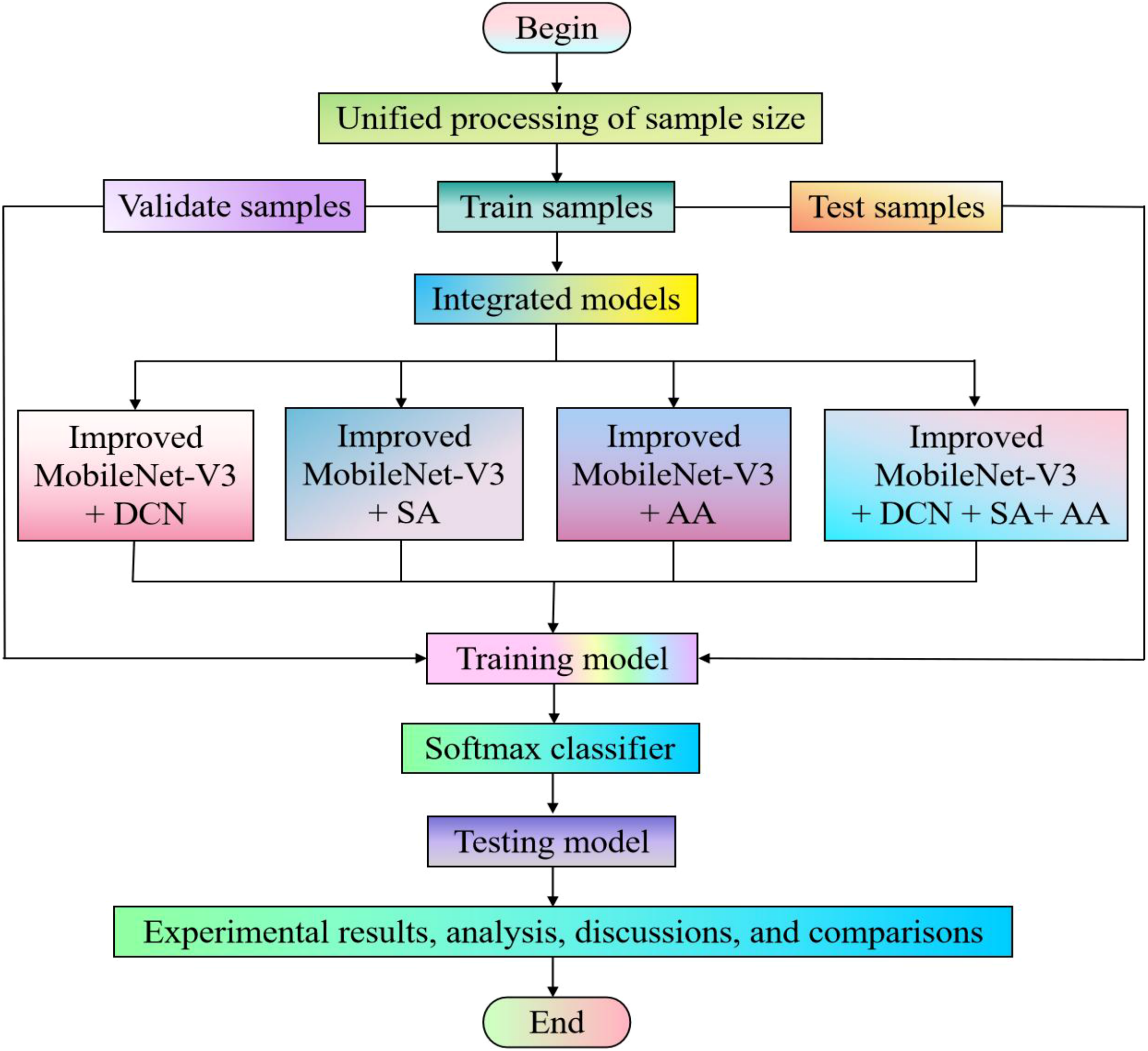
Experimental flowchart.

Due to the inconsistency in the dimensionality of feature matrix operations between convolutional and fully connected layers during data processing, when the size of the input image changes, the generated feature maps will also change accordingly, resulting in varying weights. If the weights are different after each input, the model will not be able to be trained. Therefore, most deep learning models typically require input images to have the same size. In our experiment, we used the image processing software Photoshop to batch process the training set, validation set, and test set. Based on the characteristics of the data, we uniformly adjusted the image to 224 × 224 pixels. We used identical parameter configurations for all deep learning models involved in our experiment. The specific experimental process is manifested in **Figure 6**.

To discover the selection principles for several essential parameters (e.g., batch size, learning rate, epoch, optimizer, activation function, and dropout) in the proposed model, we tested and analyzed various parameter combinations. The test results are presented in **Table 3**. **Table 3** reveals that the suggested model has varying testing accuracy and losses for different parameter combinations. When the epoch is 50 and the learning rate is 0.0002, batch size 16 has a higher test loss and accuracy than batch size 8. When epoch=50 and batch size=16, the test loss and accuracy of learning rate=0.0002 are higher than those of learning rate=0.0001. When the optimizer, activation function, and dropout are Adam, H-swift, and 0.2, respectively, test loss and test accuracy are superior to other parameter combinations. When the learning rate is 0.0002, the test loss and accuracy for epoch=50 and batch size=16 are higher than those for epoch=100 and batch size=32. Therefore, we set the batch size, learning rate, optimizer, activation function, dropout, and epoch to 16, 0.0002, Adam, H-swish, 0.005, and 50, respectively.

**Table 3:** Comparisons of different experimental configurations.

Methods	Batch size	Learning rate	Epoch	Optimizer	Activation function	Dropout	Test loss	Test accuracy
Proposed model	8	0.0002	50	Nadam	ReLu	0.1	0.4683	95.54%
	16	0.0001	50	Adam	H-swish	0.5	0.3297	94.73%
	32	0.0002	100	Nadam	ReLu	0.2	0.2971	95.89%
	8	0.0001	80	Adam	ReLu	0.3	0.4508	93.85%
	16	0.0002	50	Adam	H-swish	0.2	0.1929	99.21%
Experimental settings	16	0.0002	50	Adam	H-swish	0.2	–	–

### 4.2 Experimental data

The experimental data was sourced from a pea planting base in Henan Province, China. We used the mobile phone camera (Vivo S16e, China) to take a portion of the original image in a laboratory environment. The other part of the data is augmented by the generative adversarial network Pix2Pix. There are a total of 7915 images, including 1369 original images and 6546 augmented images. The experimental data includes one class of healthy and four classes of diseased pea leaves, namely brown spot, leaf miner, powdery mildew, and root rot diseases. There is no class imbalance among the five categories due to the small differences in quantity. The distribution of the original and augmented data for each category is displayed in **Table 4**.

**Table 4:** Distribution of each class on the original and augmented data.

Categories	Brown spot	Leaf miner	Powdery mildew	Root rot	Healthy	Total
Original data	259	271	286	298	255	1369
Augmented data	1519	1283	1054	1386	1304	6546
Total	1778	1554	1340	1684	1559	7915

**Figure 7:**
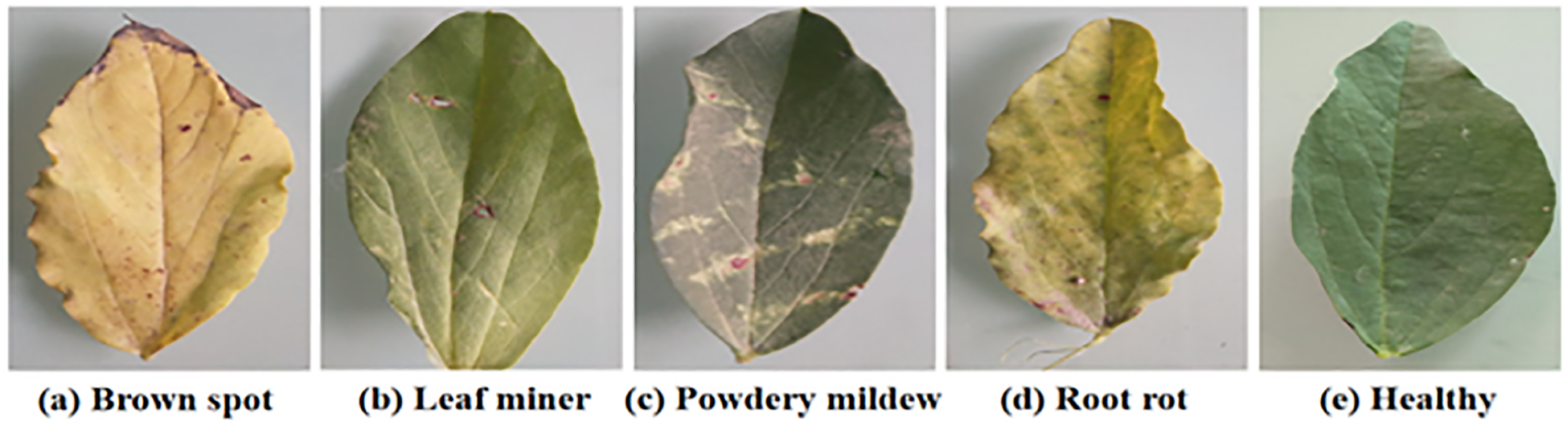
The five samples.

The five samples are shown in **Figure 7**.

The brown spot disease samples usually show brown spots with clear edges and yellow halos. The leaf miner disease samples typically exhibit white winding tunnels with dry and perforated leaves. The powdery mildew samples typically exhibit a white powdery mold layer, with yellowing and curled leaves. The root rot disease samples usually exhibit yellowing and wilting of leaves. The healthy samples typically exhibit a deep green color, smooth surface, and no spots or mold layers.

Because our research is still in its early stages, our primary goal is to swiftly evaluate the model’s viability and universality. This allows us to quickly create experimental frameworks and perform preliminary model training and evaluation. We used a 3:1:1 data allocation strategy to distribute experimental data, which takes into account the characteristics and needs of the current research stage. This segmentation method is frequently utilized in comparable deep learning model research and has some universality and referential value.

Although hierarchical k-fold cross-validation can provide more robust model evaluation results, in the current research stage, we focus more on fast iteration and the basic performance of the model. Oversampling methods may alter the original distribution of data, especially when our data volume is not particularly small. We believe that the original data distribution is more representative of practical application scenarios. We are concerned that oversampling may cause the model to learn features that do not actually exist, thereby affecting the model’s generalization ability. Therefore, we used a common deep learning dataset partition ratio of 3:1:1 to randomly divide the samples into training, validation, and testing sets. **Table 5** shows the specific quantity distribution of each category.

**Table 5:** The quantity of each category.

Categories	Training set	Validation set	Testing set	Total number
brown spot	1067	355	356	1778
leaf miner	933	311	310	1554
Powdery mildew	804	268	268	1340
Root rot	1010	337	337	1684
Healthy	935	312	312	1559
Total number	4749	1583	1583	7915

### 4.3 Train and test results

**Figure 8:**
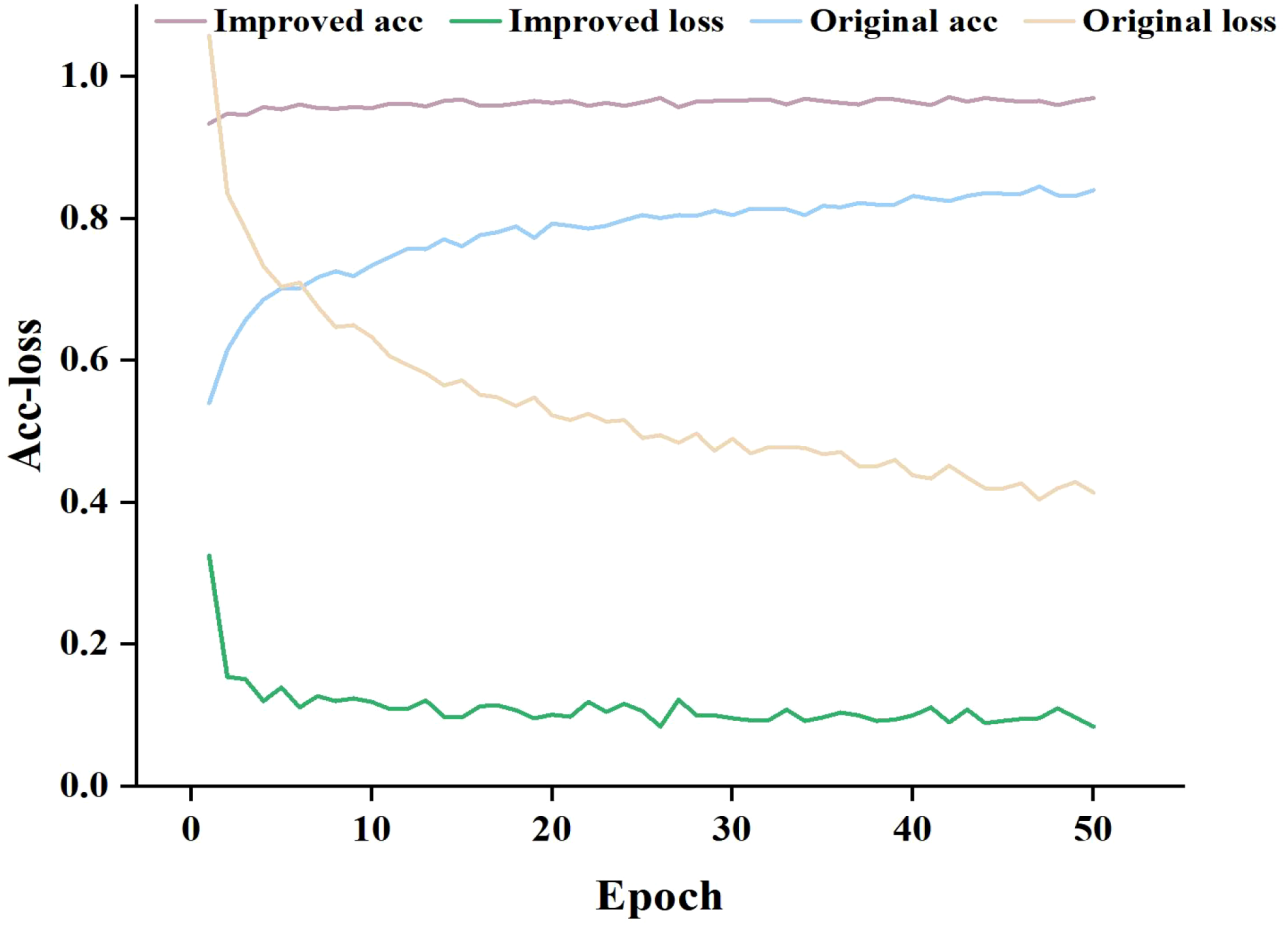
Training accuracy and loss curves.

To compare the proposed model’s feature extraction capabilities for pea leaf disease identification with the classical MobileNet-V3_small, we performed iterative training and observed the relationship between accuracy and loss value. The results are presented in **Figure 8**.

As can be seen in **Figure 8**, when the number of training epochs increases, the accuracy of both the enhanced and traditional models improves. However, in the early phases of training, the upgraded model outperforms the classical model. As training goes on, both models’ accuracy increases, but the improved model retains a lead, and the gap widens in the later stages. In terms of loss value, the trained classical model has a considerably larger loss value than the upgraded model, and the loss value of the classical model progressively stabilizes at 0.4-0.6. The loss value of the improved model finally stabilizes between 0.1 and 0.2. By comparing and analyzing the accuracy and loss value, it can be concluded that the improved MobileNet-V3_smll model has stronger feature extraction ability in pea leaf disease recognition tasks compared to the classical MobileNet-V3_small model. It can more accurately identify diseases and perform better in model fitting with lower loss values. This validates the effectiveness and superiority of the improved model.

To test several deep learning models for pea leaf disease identification, we employed specificity, precision, sensitivity, and accuracy. These indicators offer a more thorough comprehension of the model’s performance, ensuring the model’s predictive accuracy. The four evaluation metrics can be expressed Equations (13)–(17):


(13) $$Specificity=\frac{TN}{TN+TP}$$

(14) $$\mathrm{Pr}ecision=\frac{TP}{TP+FP}$$

(15) $$Sensitivity=\frac{TP}{TP+FN}$$

(16) F1−score=2×Precision×RecallPrecision+Recall

(17) $$Accuracy=\frac{TP+TN}{TP+TN+FP+FN}$$

where TP indicates that positive samples are designated as such. FP denotes that negative samples are considered positive. TN denotes that negative samples are marked as such. FN denotes that positive samples have been labeled as negative.

**Figure 9:**
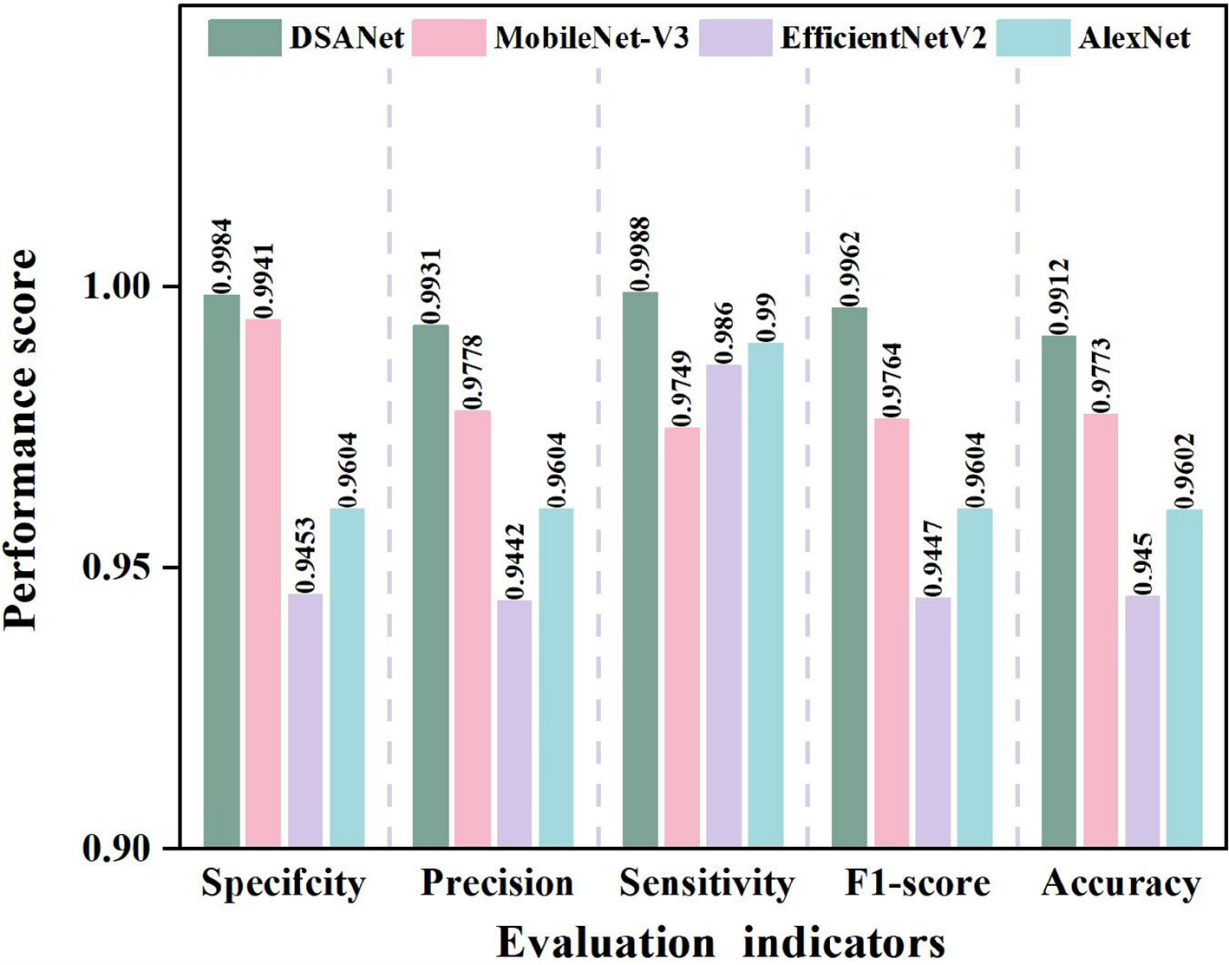
Comparison of evaluation indicators for different models.

In order to compare the identification performance of the proposed approach with other excellent deep learning models such as AlexNet, EfficientNet-v2, and the classic MobileNet-V3 for pea leaf diseases, we used specificity, precision, sensitivity, F1-score, and accuracy as evaluation metrics to draw a relevant tree diagram. The results are sketched in **Figure 9**.

Detailed information has been acquired in **Figure 9**, the suggested model performs in terms of specificity, accuracy, sensitivity, F1-score, and accuracy when compared to the other three comparison models. By avoiding the performance bottleneck or resource waste issues that EfficientNetV2 may have in some application scenarios, the DSA-Net model’s distinct structural design not only produces better results in a variety of performance indicators but may also offer advantages in computing resource consumption and other areas. At the same time, in comparison to the traditional AlexNet model, the proposed DSA-Net model successfully overcomes AlexNet’s limitations in complex feature extraction due to its relatively simple network architecture via advanced network optimization strategies, significantly improving feature extraction accuracy and comprehensiveness. Although the MobileNet-V3 model performed better, the proposed DSA-Net model outperformed it not just in terms of accuracy and other key metrics, but also in the model’s generalization capabilities and adaptability to different data sets. This finding demonstrates that the proposed DSA-Net model can efficiently harvest more important information from complex and diverse data, resulting in a more accurate and robust recognition effect.

**Figure 10:**
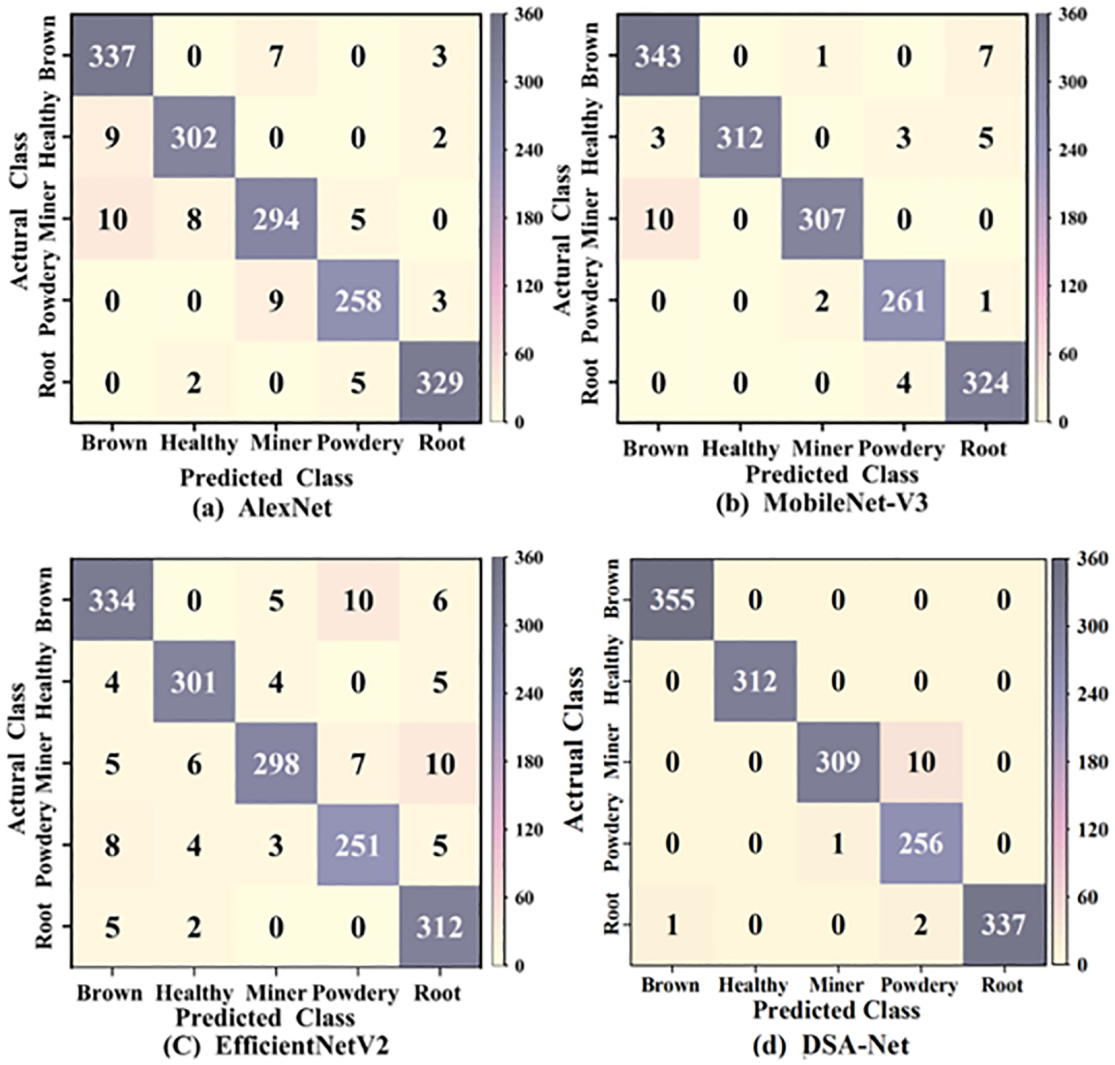
Confusion matrix classification results of different models.

A confusion matrix is a valuable tool for assessing the performance of classification models. It visibly depicts the connection between the model’s expected results and the actual labels. The rows of the confusion matrix indicate the actual category, while the columns relate to the several prediction categories. The value on the main diagonal indicates the total number of samples successfully classified. The confusion matrix allows us to properly identify the model’s weakness and guide the subsequent optimization direction. As a result, we used the confusion matrix in the image to compare the classification performance of various models for pea leaf disease recognition. The classification results of confusion matrices are presented in **Figure 10**.

As illustrated in **Figure 10**, the overall classification accuracy of the proposed DSA-Net model is 99.12%, AlexNet’s is 96.02%, EfficientNetV2’s is 94.5%, and MobileNet-V3’s is 97.73%. In comparison to other models, the proposed DSA-Net model outperforms EfficientNetV2 by 4.62% while maintaining the lowest accuracy. The proposed DSA-Net model has a high identification rate for health, and all categories are correctly predicted. Powdery mildews have the lowest classification rate. The proposed model has weak recognition ability for the powdery mildew disease. At the feature level, the background contour of powdery mildew is more complex and diverse than that of the leaf miner disease. The disease characteristic area of powdery mildew is more extensive than that of leaf miner. The complexity of the background contour of powdery mildew and the wide range of disease areas have weakened the recognition ability of the proposed model. From a color perspective, leaf miners form white winding tunnels when larvae feed on leaf flesh, and the tunnels may turn white later due to oxidation. And the surface of powdery mildew leaves is covered with a white powdery mold layer. The variation of diseases with the same color reduces the recognition ability of the model. From a morphological perspective, leaf miners can experience leaf curling and deformation due to larval feeding and powdery mildew infection by fungi. The same curling and deformation of leaves also increase the difficulty of identifying powdery mildew in the model. In summary, the proposed DSA-Net model performs well in the task of recognizing pea leaf diseases.

In deep learning research, an ablation experiment is a method for determining the requirement and effectiveness of each model component. We can assess the contribution of a model component by progressively removing or altering it and seeing how its performance changes. Because the suggested approach combines deformable convolution, self-attention, and additive attention mechanisms.

**Figure 11:**
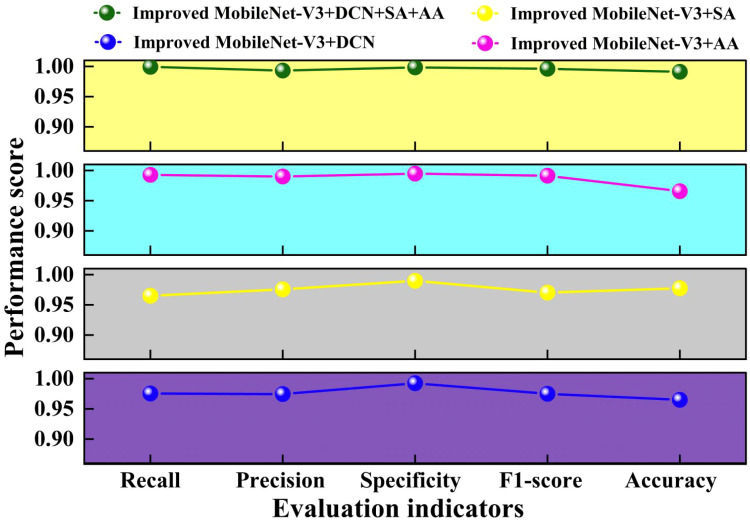
Comparison of different evaluation indicators in ablation study.

To further validate the feasibility of the ablation study, we tested different ablation combinations using four different evaluation metrics. This result is sketched in **Figure 11**.

From **Figure 11**, it can be seen that the four evaluation metrics of the proposed Improved MobileNet-V3+DCN+SA+AA method are higher than the other three ablation experimental methods. Compared with the lowest Improved MobileNet-V3+SA, Improved MobileNet-V3+DCN+SA+AA increased by 0.86%~3.43%. Compared with the highest Improved MobileNet-V3+SA, Improved MobileNet-V3+DCN+SA+AA increased by 0.3%~2.56%. This indicates that the attention mechanism can filter and weight the features extracted by deformable convolution, highlight the key features of the target, suppress irrelevant information, better cope with the deformation and scale changes of the target object, and improve model performance. Adding AA alone to the improved MobileNet-V3 is slightly higher than adding SA alone. Adding DCN alone to the improved MobileNet-V3 results in slightly higher evaluation metrics than adding SA alone. This indicates that introducing DCN in layers 3, 4, and 8 can to some extent explore deep and potential feature interaction patterns in the data, improving the performance of the model. layers3, Layers 4 and 8 play a crucial role in feature transformation and expression in the model. Introducing DCN can effectively improve the performance of the model without significantly increasing the overall computational complexity. Adding AA alone on the improved MobileNet-V3 is slightly higher than adding DCN alone. This indicates that adding AA alone on the improved MobileNet-V3 can improve the performance of the model more than adding DCN and SA alone. Overall, the Improved MobileNet-V3+DCN+SA+AA model performs well in all evaluation metrics, demonstrating its superiority in pea leaf disease recognition tasks.

**Figure 12:**
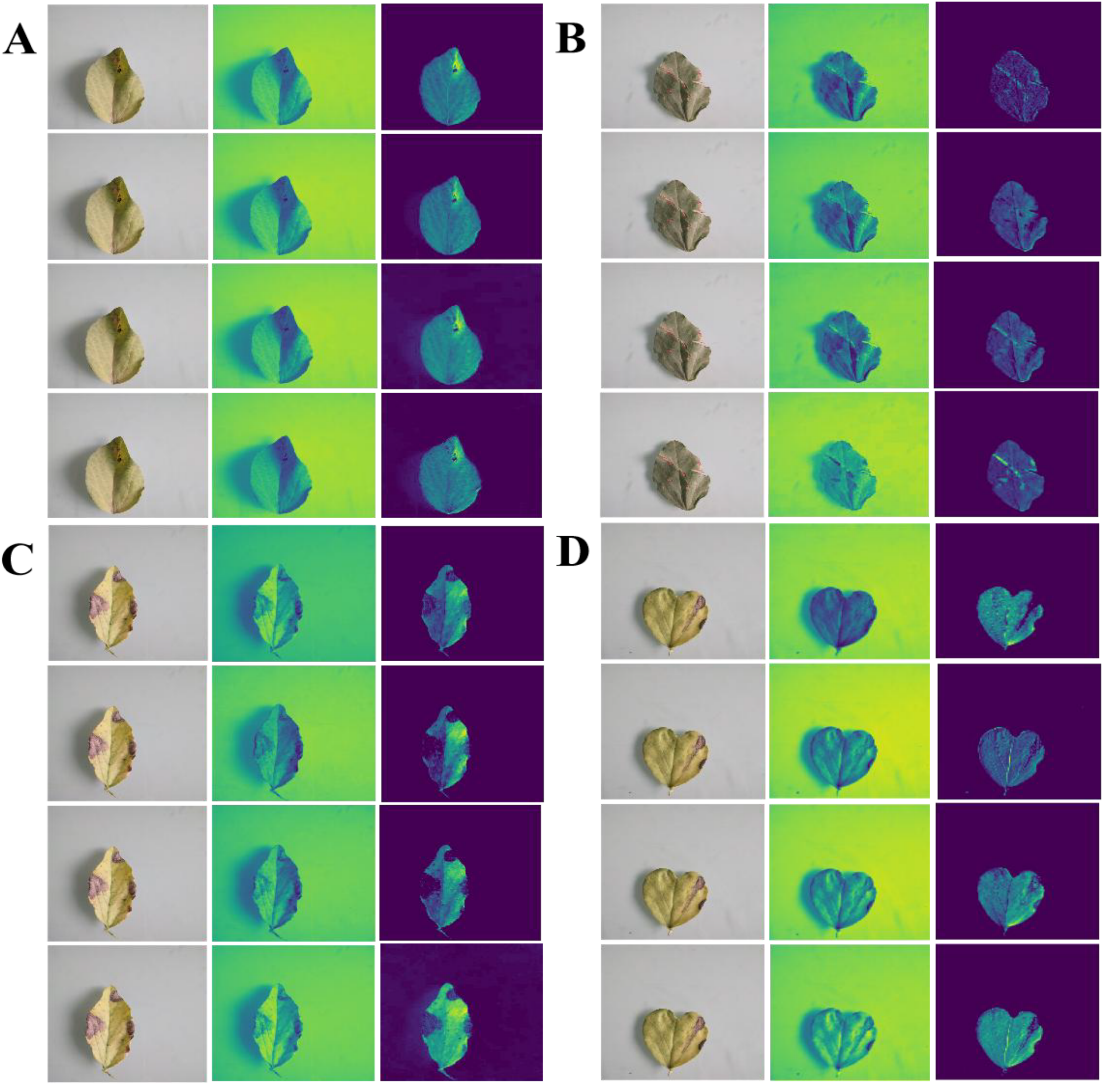
Visual heatmap of pea leaf disease feature extraction under different ablation experiments **(A–D)** reported Improved MobileNet-V3+DCN, Improved MobileNet-V3+SA, Improved MobileNet-V3+AA, Improved MobileNet-V3+DCN+SA+AA Four methods are used from top to bottom to extract heatmaps of leaf diseases for root rot, powder mill, brown spot, and leaf miner.

To further validate the feature extraction of the proposed ablation method for four pea leaf diseases, we visualized the leaf disease feature extraction under different methods using a heatmap. This result is displayed in **Figure 12**.

**Figure 12** shows that when the SA is added to the enhanced MobileNet-V3, the heatmap’s response to the lesion area is somewhat distributed, the highlight heat is not focused, and the effect of collecting small spots is not optimal. When the DCN is introduced to the improved MobileNet-V3, the activation intensity of the heatmap at the center of the lesion increases dramatically, but edge localization remains imprecise. When the AA module is introduced to the enhanced MobileNet-V3, the spatial dimension stores positional information more effectively, and the heatmap highlights are distributed more uniformly throughout the lesion’s edge. Adding a separate AA module can better capture the characteristics of disease areas than adding separate DCN and SA modules. When the DCN, SA, and AA are combined with the enhanced MobileNet-V3, the extracted heatmap reveals the most concentrated highlight effect in the lesion area, particularly in minor lesions and edges. This shows that the three modules we introduced at the same time not only improve the accuracy of lesion localization and the complementary gain of feature expression, but they also do not interfere with one another or influence classification performance.

**Figure 13:**
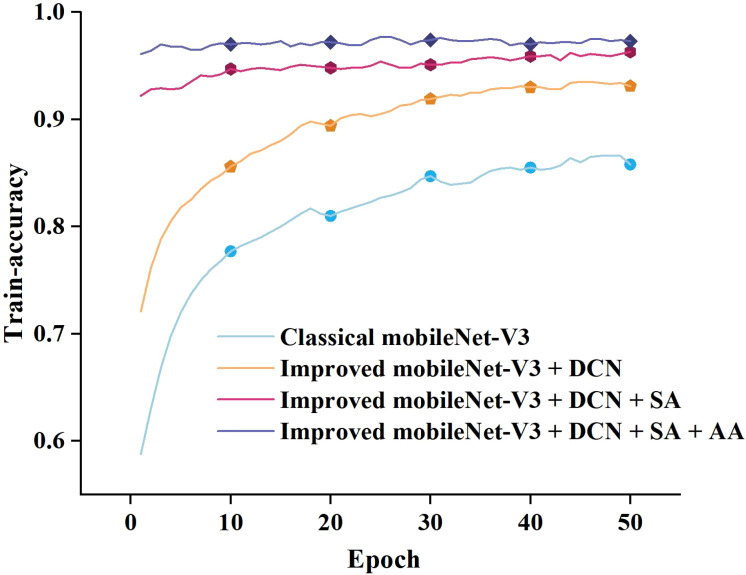
The results of ablation experiments on our dataset.

We employed the identification accuracy as a comparative standard. The ablation experiment results of different model combinations are depicted in **Figure 13**.

As illustrated in **Figure 13**, when only the classical model is utilized, the accuracy rate gradually increases with the number of training rounds. After introducing the deformable convolution neural (DCN) module, the model’s accuracy rate improves dramatically and eventually stabilizes at a high level. When the self-attention mechanism is added to this, the accuracy rate improves dramatically and remains rather consistent during the training period. After adding deformable convolution, a self-attention mechanism, and an additive attention module to the classical model, the accuracy rate increased and remained near 1.0 during the training period, indicating that the module improved the model’s performance. In general, the model’s accuracy improved gradually as DCN, self-attention, and additive attention mechanisms were added. This demonstrates that the combination of these modules can successfully increase model performance, with each module contributing to the model’s accuracy to varied degrees.

**Figure 14:**
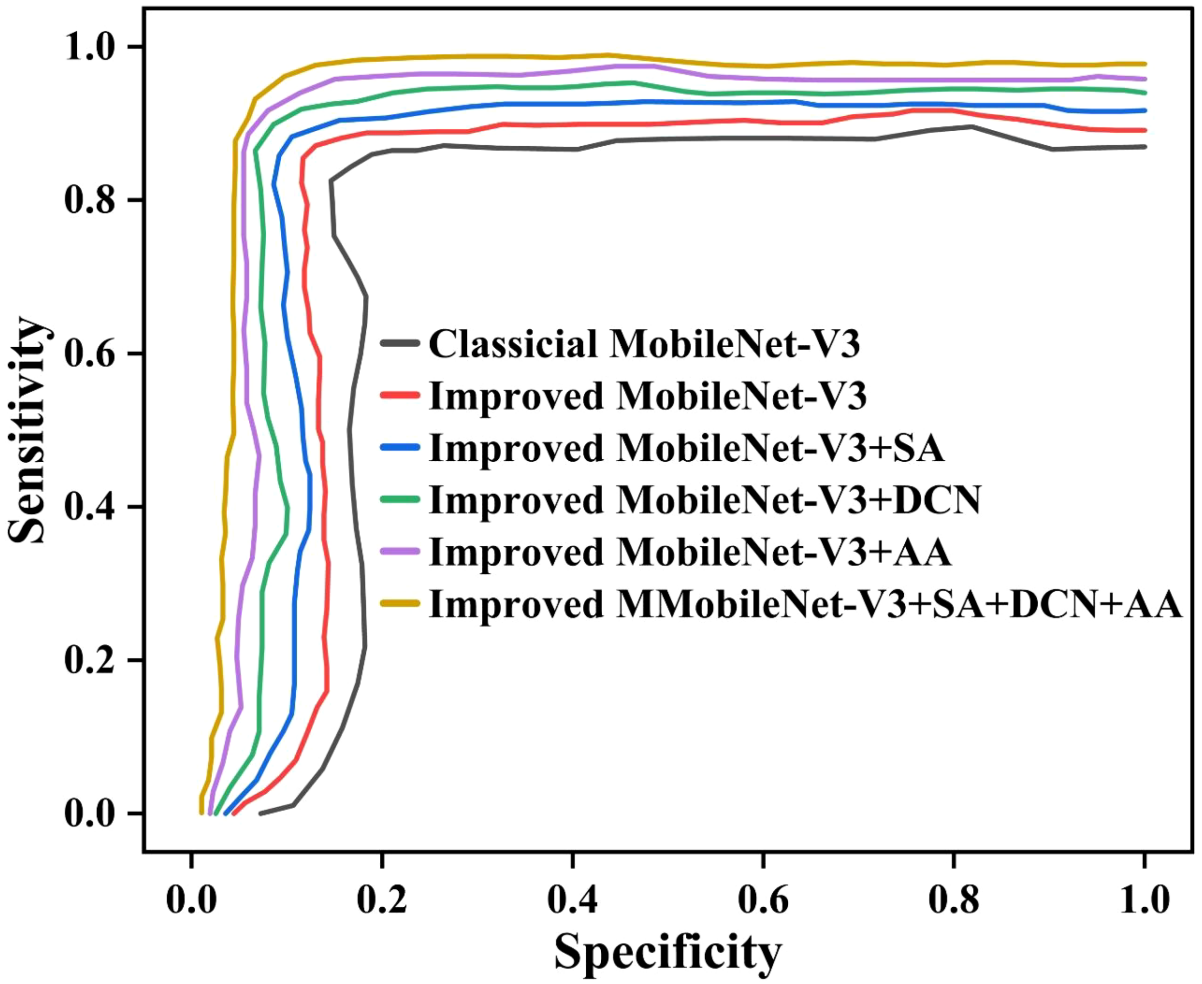
The ROC curves of different ablation study methods.

To comprehensively evaluate the performance of the classic MobileNet-V3 and its improved versions in identifying pea leaf diseases, especially considering the bias that may be caused by imbalanced class distribution. We have plotted the receiver operating characteristic (ROC) curves of each model in **Figure 14**. The ROC curve is plotted with sensitivity as the Y-axis and specificity as the X-axis. It presents a performance comparison between the classical MobileNet-V3 and improved models Improved MobileNet-V3, Improved MobileNet-V3+SA, Improved MobileNet-V3+DCN, Improved MobileNet-V3+AA, and Improved MobileNet-V3+SA+DCN+AA.

From **Figure 14**, it can be seen that the ROC curves of the six models show a steep upward trend in the high sensitivity region (close to 1.0), but there are differences in the curves when the 1-specificity is low (0.0 to 0.2). This indicates that the model has strong recognition ability for most categories, but its performance for a few categories may be limited by imbalanced class distribution. Especially the classical MobileNet-V3 curve shows a significant increase in the low 1-specification region. This indicates that it has weak adaptability to imbalanced data. This is due to the uneven distribution of sample size, which leads to an increase in misclassification of minority categories. The proposed Improved MobileNet-V3+SA+DCN+AA exhibits higher sensitivity across the entire 1-specification range, with its curve closer to the upper left corner. This indicates that combining the SA, DCN, and AA can effectively alleviate the impact of distribution imbalance and enhance the model’s ability to distinguish minority categories. Especially when the 1-specification is below 0.1, the performance of the curve is better than that of the classical MobileNet-V3. It reflects the improved robustness of the model under high specificity conditions. The unbalanced distribution significantly affects the performance of the classical MobileNet-V3, and the improved model alleviates this problem through structural optimization.

**Figure 15:**
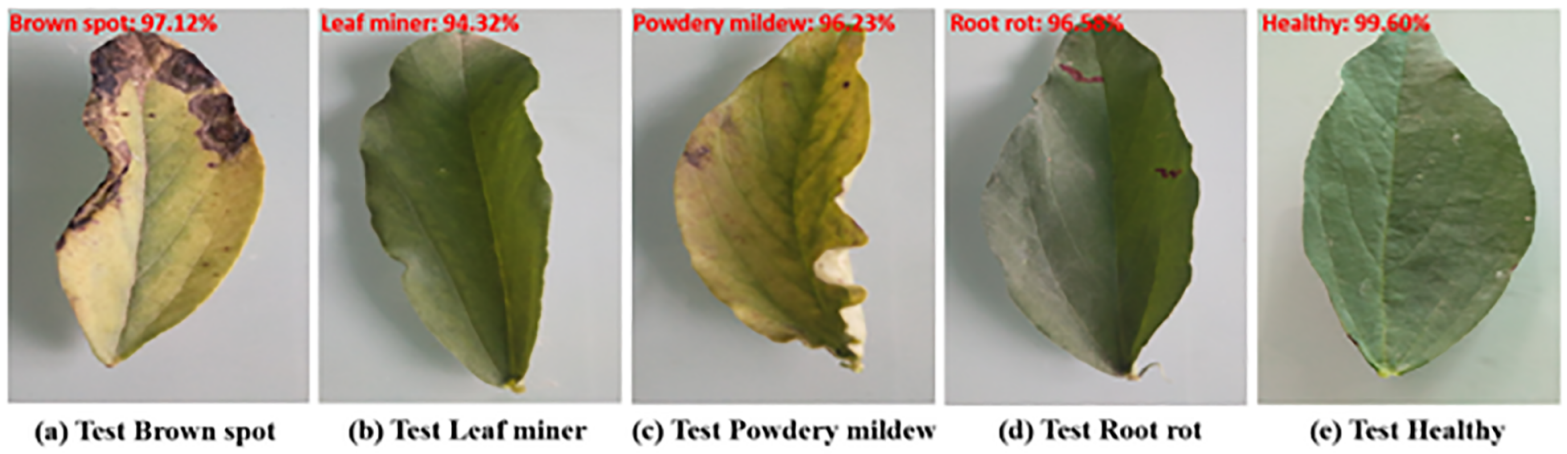
The test results on our test dataset.

We randomly selected four samples from the test set and used the proposed method to test them. The predicted results are presented in **Figure 15**. All predictions were correct and the recognition probability was above 94.00%.

We tested the suggested method for recognizing distinct plant leaf diseases against various open-source plant leaf disease data to ensure its generality, class imbalance, and robustness. **Table 6** lists this outcome.

**Table 6:** Comparison of test results for different plant leaf diseases.

Dataset names	Recall	Precision	F1-score	Specificity
Our own pea leaf disease data	99.94%	99.31%	99.62%	99.84%
Plant Pathology Apple Dataset	99.80% (↓0.14%)	99.40% (↑0.09%)	99.60% (↓0.02%)	99.90% (↑0.14%)
New Plant Diseases Dataset (Potato)	99.70% (↓0.24%)	99.50% (↑0.19%)	99.60% (↓0.02%)	99.80% (↓0.04%)
PlantifyDr Dataset (Corn)	99.90% (↓0.04%)	99.20% (↓0.09%)	99.55% (↓0.07%)	99.85% (↓0.01%)
Rice Diseases Image Dataset	99.85% (↓0.09%)	99.35% (↑0.04%)	99.60% (↓0.02%)	99.75% (↓0.09%)

**Table 6** indicates that the proposed model outperforms four previous open-source plant leaf disease datasets. In terms of recall and F1-score, the suggested model offers equivalent evaluation metrics for all five categories of data. In terms of precision, the proposed model outperforms the PlantifyDr Dataset (Corn) by 0.09% on its own data. In terms of specificity, the proposed model is 0.14% lower on its own data compared to the PlantPathology Apple Dataset. Compared with the results obtained from our own pea leaf disease data, some indicators on these open-source datasets have slight fluctuations, indicating that the class imbalance of data collected under different environmental conditions poses certain difficulties to the classification ability of the model. Overall, our method still maintains high robustness and universality in identifying different plant leaf diseases.

We collected the latest literature on plant leaf diseases. The results are displayed in **Table 7**.

**Table 7:** Comparison of the proposed method with other recent literature.

Authors., Ref., Year	Methods	Dataset size	Number of Classes	Category	Accuracy
Ruby et al. (2024)	Modified ResNet50	4500	4	Wheat	98.44%
Shoaib et al. (2022)	Modified U-Net	18161	10	Tomato	99.35%
Xiang et al. (2025)	DWTFormer	54306	9	Tomato	99.28%
Bhavani (2025)	Convolutional autoencoder	1166	5	Soybean	92%
Seelwal et al. (2024)	Deep learning	5932	6	Rice	94.25%
Alkanan and Gulzar (2024)	MobileNetV2	17,801	4	Corn	96%
Gulzar (2024)	Improved Inceptionv3	5513	5	Soybean	98.73%
Gulzar et al. (2025a)	Transfer learning	1214	3	Alfalfa	99.45%
Gulzar and Ünal (2025b)	PL-DenseNet	3505	4	Pear	99.18%
Gulzar and Ünal (2025c)	PlmNet	400	3	Plums	97.58%
Proposed. (2025)	DSA-Net	7915	5	Pea	99.12%

From **Table 7**, it can be seen that the proposed model improves by 7.12% compared to the lowest convolutional autoencoder network proposed by Bhavani (2025). Compared with the transfer learning proposed by Gulzar et al. (2025a), the proposed model reduces by 0.33%. The classification performance of the proposed model, PL DenseNet, Modified U-Net, and DWTFormer is not significantly different, ranging between 99.12% and 99.35%. The classification performance of Modified ResNet50 and Improved Inceptionv3 is not significantly different, both between 98.44% and 98.73%. Overall, the proposed model performs better than most and lower than a few plant leaf disease classification models. This indicates that the proposed model has high recognition performance for pea leaf diseases.

### 4.4 Deployment potential on edge devices

We first co-designed the DCN, SA, and AA modules in the lightweight model MobileNet-V3 and achieved an accuracy of 99.12% on 5 types of pea leaf disease data. It outperforms the EfficientNet and ShuffleNet models while maintaining a good 1.48M parameter count for edge deployment. To further validate the rationality of the proposed model’s inference speed, robustness, and feasibility of edge deployment. We tested the params, FLOPs, speed, and accuracy of four lightweight models with 32g of memory and an RTX 4090 graphics card. The specific results are presented in **Table 8**.

**Table 8:** Comparison of the proposed method with other methods.

Models	Params (M)	FLOPs (G)	Speed(ms)	Accuracy
MobileNet-V3	1.73	3.26	1.94	97.73%
ShuffleNetv2	1.45	4.08	2.09	96.02%
EfficientNetV2	2.07	3.96	1.89	94.50%
DSA-Net	1.48	2.65	1.52	99.12%

The proposed DSA-Net outperforms the other three lightweight models in terms of FLOPs, speed, and accuracy. On Params, the proposed DSA-Net is 0.03M more than the highest EfficientNetV2. In FLOPs, the proposed DSA-Net is 2.65GB less than the highest ShuffleNetv2. On Speed, the proposed DSA-Net is 1.52 ms faster than MobileNet-V3. The proposed DSA-Net is significantly better than the other three models in terms of accuracy. The parameter quantities of ShuffleNetv2 and the proposed DSA-Net are not significantly different, indicating that they have achieved a good balance between model performance and computational resource consumption. The proposed DSA-Net exhibits significant advantages in both accuracy and inference speed. Although slightly higher in parameter count than ShuffleNetv2, its overall performance is within a reasonable range. It achieves a good balance between model performance and computational resource consumption. This fully validates its feasibility and superiority in edge deployment scenarios.

The suggested method has great potential in edge device deployment. MobileNet-V3 itself is an efficient, lightweight model designed to meet resource-constrained scenarios such as edge computing. It has the characteristics of fewer parameters and lower computational complexity. On the basis of MobileNet-V3, we have carefully designed and optimized the integration of deformable convolution, self-attention mechanism, and additive attention mechanism for the first time. Although deformable convolution generates additional offset learning, it effectively controls computational overhead through reasonable parameter sharing and optimization strategies. The self-attention mechanism and additive attention mechanism adopt efficient computational methods in the collaborative implementation process, avoiding excessive computational costs. Therefore, the proposed model still maintains its lightweight characteristics. This is conducive to deployment on edge devices with limited computing resources and small memory capacity so as to realize real-time processing of images and videos, meet the requirements of edge computing for low latency and low power consumption, and provide a reliable reference technology scheme for intelligent applications on edge devices.

In the future, to further enhance the edge deployment of the proposed model, we will carry out work from multiple aspects. In terms of model compression, we plan to use techniques such as knowledge distillation and quantification to optimize the model. Because knowledge distillation can transfer knowledge from complex models to smaller models. It can further reduce the number of model parameters while maintaining high performance. Quantitative approaches can transform floating-point parameters in a model to low-precision integer parameters, decreasing the model’s storage and processing requirements while also making it more suited for edge device hardware. In terms of hardware configuration, we will conduct in-depth research on the hardware architecture and computing characteristics of different edge devices and optimize and adjust the model accordingly. For example, for edge devices with specific acceleration units, we will optimize the computational flow of the model, fully utilize the parallel computing capabilities of hardware, and improve the running efficiency of the model. Furthermore, we will investigate the model’s block deployment strategy, distribute various model components to various edge devices or edge cloud collaboration systems, accomplish load balancing, enhance the system’s overall performance and stability, and make our model more applicable in a greater variety of edge computing scenarios.

## 5 Conclusions

In this study, we provide a pea leaf disease detection technique based on fused dual attention processes and enhanced MobileNet-V3. With an average recognition accuracy of 99.12% on the test set, the suggested approach can successfully enhance the model’s capacity to extract and identify the traits of pea leaf diseases. The proposed DSA-Net outperforms the lightweight models EfficientNet and ShuffleNet while retaining a sufficient amount of parameters for edge deployment (1.48M), less FLOPs (2.65g), and quicker speed (1.52ms)., PlantifyDr Furthermore, the suggested model achieved 99.9% recall (e.g., PlantifyDr Dataset), 99.5% precision (e.g., PlantPathology Apple Dataset), 99.6% F1 score (e.g., PlantifyDr Dataset and Rice Diseases Image Dataset), and 99.8% specification (e.g., New Plant Diseases Dataset) on four open-source datasets. Even though the suggested approach has produced some positive outcomes, there are still certain issues that require improvement and refinement:

(1) Optimize attention mechanism fusion strategy: Although the proposed strategy sequentially integrates deformable convolution, self-attention, and additive attention mechanisms and achieves certain results, this fusion method is not optimal. The synergistic effect of different attention mechanisms has not been fully explored. In the future, we will study the fusion methods and synergistic effects between different attention mechanisms to obtain more efficient fusion strategies.

(2) Improving model interpretability: Although the proposed model has achieved good results in disease recognition tasks, there is still a lack of in-depth explanation on how the model makes decisions and which features play a key role in the recognition results. In the future, we will conduct research on the interpretability of deep learning models, using visualization, feature importance analysis, rule extraction, and other methods to reveal the decision-making process of the models.

(3) Optimize the model: In the process of improving the model, we focus on maintaining its lightweight characteristics, but with the introduction of multiple mechanisms, the complexity of the model has increased, and the demand for computing resources has correspondingly increased. In the future, we will use model pruning, quantization, and knowledge distillation to remove redundant parameters and structures in the model and improve the real-time performance of the model on mobile devices and embedded systems. On the other hand, we will explore more efficient neural network architecture design methods that balance model performance and computational efficiency as much as possible while ensuring model performance.

## Data Availability

The original contributions presented in the study are included in the article/supplementary material. Further inquiries can be directed to the corresponding author.
